# Synthesis and Tribological Characteristics of High-Performance Self-Lubricating CoCrFeNiMo_x_-Ni/MoS_2_-Ag-Cr_2_O_3_ Composites

**DOI:** 10.3390/ma19040783

**Published:** 2026-02-18

**Authors:** Bei Sun, Zhiming Gao, Zhongtang Gao

**Affiliations:** 1 College of Aviation Maintenance Engineering, Xi’an Aeronautical Polytechnic Institute, Xi’an 710089, China; 2 School of Mechanical Engineering, Xi’an University of Science and Technology, Xi’an 710054, China

**Keywords:** CoCrFeNi, high entropy alloy, spark plasma sintering, wear, tribo-chemistry

## Abstract

High-temperature self-lubricating materials with stable tribological performance across a wide temperature range are essential for advanced mechanical systems under extreme conditions. However, balancing mechanical strength and lubrication efficiency remains a key challenge. This study fabricated CoCrFeNiMox-Ni/MoS_2_-Ag-Cr_2_O_3_ composites (*x* = 0.2, 0.5, 1) via spark plasma sintering, aiming to investigate the effect of Mo content on their microstructure, mechanical properties, and tribological behavior. Microstructural analysis showed that the as-sintered composites mainly consist of FCC phase, Cr_2_O_3_, Ag, and Ni/MoS_2_. Increasing Mo content from 0.2 to 1 wt.% significantly promoted the formation of hard σ-phase intermetallics, leading to increased hardness (up to 546 HV) and yield strength (peaking at 502 MPa). Tribological tests at 25–800 °C indicated continuous lubrication behavior in all composites. The minimum friction coefficient was 0.23, and wear rates remained below 10^−6^ mm^3^/N·m. In the low-to-medium temperature range, lubrication was dominated by the synergistic effect of Ni/MoS_2_ and Ag: Ni/MoS_2_ formed low-shear-strength films, while Ag reduced surface adhesion. Meanwhile, the Mo solid solution strengthened and the σ-phase enhanced wear resistance by improving hardness and inhibiting plastic deformation. At high temperatures, tribochemical reactions generated lubricating films composed of oxides and molybdates, which maintained tribological performance by reducing direct contact between friction pairs. This study demonstrates that Mo-doped high-entropy alloy composites can serve as high-performance wide-temperature self-lubricating materials, providing a basis for designing “matrix-lubricant” systems for extreme-temperature applications.

## 1. Introduction

The rapid development of aerospace, nuclear industries, and new energy vehicles demands effective lubrication and wear control for critical moving components, as these directly determine the performance and operational lifespan of machinery. Achieving a low coefficient of friction (COF) and wear rates (WR) under extreme conditions such as high temperature, heavy load, and irradiation environments remains a persistent challenge in tribology [1,2,3]. Solid lubricants can effectively separate surfaces while providing lubrication and wear resistance. However, most solid lubricants suffer from thermal instability at elevated temperatures: graphite undergoes oxidative decomposition above 450 °C and MoS_2_ transforms into non-lubricious MoO_3_ beyond 500 °C [4]. Therefore, lubricant hybridization and tribochemically modified compositions offer promising approaches for extreme conditions such as wide-temperature-range operation [5].

Mo, Ag, and MoS_2_ are often used as additives in composites. These additives can form lubricating oxides and molybdates such as MoO_3_ and Ag_2_MoO_4_ on the worn surfaces of composites at elevated temperatures [6,7]. Hector Torres et al. investigated the high-temperature lubrication properties of various oxides and molybdates and found that MoO_3_ (0.20), NiMoO_4_ (0.29) and Ag_2_MoO_4_ (0.28) exhibit low COF at 704 °C [4,8]. Wang et al. studied the tribological behavior of Ni_3_Al-Ag based self-lubricating alloys in a wide temperature range and revealed a wear mechanism in which Ag provides lubrication at lower temperatures, while tribochemically generated NiO and Ag_2_MoO_4_ contribute to friction reduction at higher temperatures [9]. The combination of Ag and Ag_2_MoO_4_ exerts a synergistic effect, enabling excellent lubrication over a broad temperature range. Enkang Hao et al. examined the high-temperature tribological characteristics of NiCoCrAlYTa/Ag/Mo coatings and demonstrated that the outward diffusion and oxidation of Ag and Mo at high temperatures lead to the formation of a continuous β-Ag_2_MoO_4_ lubricating film on the coating surface [10]. Meanwhile, minor hard particles such as NiO within the coating effectively bear the load during friction, resulting in optimal tribological performance of the composite coating. Considering the lubricating oxides and molybdates formed on worn surfaces under high-temperature conditions, it is of great significance to pre-design self-lubricating composites to control the high-temperature friction and wear performance [11].

High-entropy alloys (HEAs) are novel alloy systems with outstanding properties demonstrating considerable potential in practical applications [12,13]. Research on CoCrFeNi HEAs has mainly focused on their microstructure, mechanical properties, and oxidation resistance, while investigations into their tribological performance over a wide temperature range remain limited [14,15]. Zhang et al. fabricated two distinct CoCrFeNi-based self-lubricating composites via spark plasma sintering (SPS). Their study revealed that the composites exhibit excellent mechanical properties and favorable friction and wear performance from room temperature (RT) to 800 °C, which is attributed to the synergistic effects among the HEA matrix, solid lubricant phases, and various metal oxides generated on the worn surface through tribochemical reactions [16,17].

To address high-temperature lubrication challenges in industrial applications, this work employed CoCrFeNi HEAs as the matrix, with Cr and Mo as strengthening elements and Ag and Ni/MoS_2_ as lubricating phases. CoCrFeNiMo_x_-Ni/MoS_2_-Ag-Cr_2_O_3_ composites (*x* = 0.2, 0.5, 1) were fabricated by SPS and designated Mo0.2, Mo0.5 and Mo1. This study investigated the microstructure, hardness, yield strength, and tribological behavior (RT—800 °C) of the composites, providing a valuable reference for designing HEA-based self-lubricating composites for applications over a wide temperature range.

## 2. Materials and Methods

The self-lubricating CoCrFeNiMo_*x*_-Ni/MoS_2_-Ag-Cr_2_O_3_ composites (*x* = 0.2, 0.5, and 1) were formulated with the following constituent ratios: 67.5 wt.% CoCrFeNiMo_x_ matrix, 12.5 wt.% Ni/MoS_2_ composite powder, 15 wt.% metallic Ag phase, and 5 wt.% Cr_2_O_3_ ceramic phase.

The composites were prepared by power metallurgy. A planetary ball mill was used to grind the materials at 150 r/min for 8 h to obtain uniform mixed powders. The mixed powders were loaded into a graphite mold, and then sintered by SPS under vacuum conditions. The sintering process was divided into three stages: (1) heating from RT to 1250 °C with the heating rate of 150 °C/min; (2) holding at 1250 °C for 3 min; (3) cooling with the furnace. The sintering process was conducted under vacuum conditions and a pressure of 40 MPa.

The bulk composite was characterized by X-ray diffraction (XRD, Bruker D8 ADVANCE, Bruker Corporation, Billerica, MA, USA) to determine the phase composition, with a test voltage of 40 kV and a scan rate of 2 deg/min. Then, a scan electron microscope (SEM, JSM-7610F) and energy dispersive spectroscopy (EDS, Thermo Scientific UItraDry, Waltham, MA, USA) were used to investigate the microstructure and chemical compositions of the samples.

The hardness was measured using an HV-1000 type Vickers hardness tester (Laiwu Express Measuring Tools & Instruments Co., Ltd., Jinan, China), with a load of 300 g and a holding time of 10 s. The compression properties were tested on a CMT5202 material testing instrument, with sample dimensions of *Φ* 4 mm × 8 mm and a testing rate of 1 mm/min.

The tribological performance were evaluated using a ball-on-disk high temperature tribometer (HT-1000, Lanzhou Zhongke Kaihua Technology Development Co., Ltd., Lanzhou, China) in the atmosphere at RT, 200, 400, 600 and 800 °C. The disk blocks were composed of Mo0.2, Mo0.5 and Mo1 composites with dimensions of *Φ* 30 mm × 5 mm, and the couple ball was made of Si_3_Ni_4_ with a diameter of *Φ* 6 mm. The wear test was conducted under the following conditions: a load of 8 N, a rotation radius of 4 mm, a sliding speed of 0.15 m/s, and a sliding distance of 270 m. After the test, the worn surfaces were analyzed by SEM and EDS. The high temperature tribometer automatically collected and recorded the coefficient of friction (COF). The wear rate (WR mm^3^/N.m) was calculated by the formula *w* = *V*/*FS*, where *V* is the wear volume (mm^3^), *S* is the total sliding distance (m), and *F* is the normal load (N). The wear volume (V) was obtained by the non-contact surface profiler (Micro-XAM-3D, Schneider GmbH & Co. KG, Höhr-Grenzhausen, Germany). The oxide composition on the worn surface of Mo1 composites at 800 °C was further determined by laser Raman spectroscopy (Thermo, Waltham, MA, USA).

## 3. Results and Discussion

### 3.1. Material Characterization

**Figure 1:**
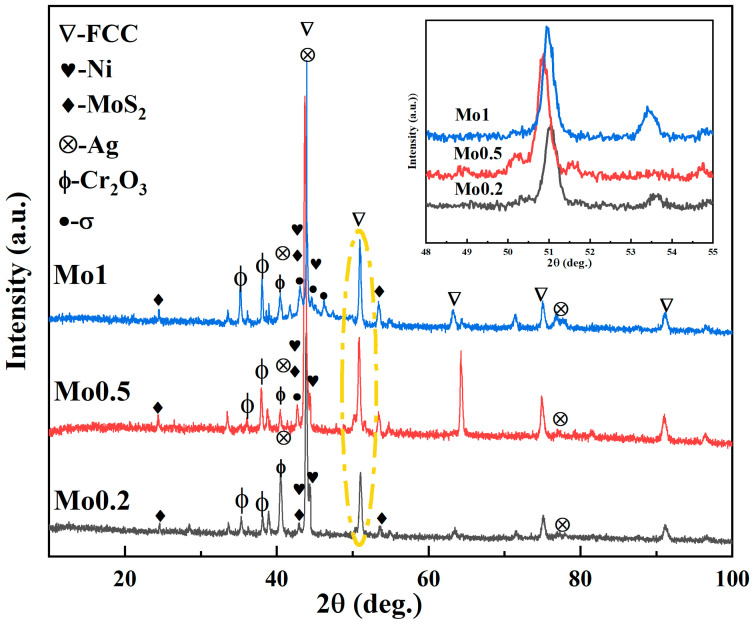
XRD patterns of Mo0.2, Mo0.5 and Mo1.

To analyze the phase composition of composites with different Mo concentrations, XRD characterization was conducted. Figure 1 shows the XRD patterns for different Mo additions. The patterns indicate that Mo0.2 has an FCC lattice and consists of Cr_2_O_3_, Ag and Ni/MoS_2_. The matrix alloy primarily forms FCC solid solution, which is attributed to the comparable atomic sizes of Co, Cr, Fe and Ni, the negligible positive or negative atomic pair mixing enthalpies among these elements, and high configurational entropy that effectively lowers the Gibbs free energy [12]. Although the FCC solid solution is observed in Mo0.2, increasing the Mo concentration results in the detection of σ-phase peaks near FCC phase peaks in Mo0.5 and Mo1. The σ phase has a tetragonal structure with lattice parameters of a = 9.2443 Å, c = 4.7921 Å and c/a = 0.5183. The lattice constants for Mo0.2, Mo0.5, and Mo1, calculated using the Scherrer equation, are 3.5573 Å, 3.5796 Å and 3.5878 Å, respectively.

As shown in the locally magnified image within the yellow dashed box in Figure 1, the diffraction peaks of Mo0.5 shift towards smaller 2θ angles compared to those of Mo0.2. This shift arises because the atomic radius of Mo (1.39 Å) is larger than that of the other elements (Co: 1.25 Å, Cr: 1.28 Å, Fe: 1.26 Å, Ni: 1.24 Å). The incorporation of Mo as a solute atom into the FCC matrix induces lattice distortion. In contrast, the diffraction peaks of Mo1 shift towards larger 2θ angles compared to those of Mo0.5. The reason is that when the addition amount of Mo is high, the lattice distortion effect caused by solution strengthening of Mo atoms is consequently greater than the lattice contraction effect induced by precipitated phase, and Mo1 still exhibits significant lattice distortion [18]. At the same time, according to the mixing enthalpy of different atomic pairs calculated by the Miedema model, the mixing enthalpy between Mo, Cr and other elements is relatively negative, and it is easy to form intermetallic compounds [19]. When the content of Mo element is low, the high entropy effect caused by multiple principal elements effectively inhibits the phase separation of the alloy, and the alloy is a single solid solution. When the content of Mo element continues to increase, and the solid solubility of Mo in the matrix phase is limited, the increase in mixing entropy is not enough to offset the effect of mixing enthalpy, so intermetallic compounds are formed, and their proportion is increasing.

**Figure 2:**
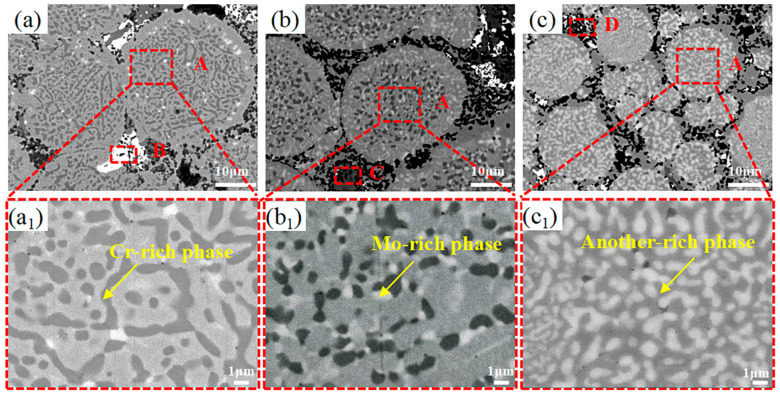
SEM images of Mo0.2 (**a**,**a_1_**), Mo0.5 (**b**,**b_1_**) and Mo1 (**c**,**c_1_**).

As shown in Figure 2a–c, there are few pores or other defects in the microstructure of the sintered composites, indicating that the composites are nearly fully densified. Ag (region B), Ni/MoS_2_ (region C) and Cr_2_O_3_ (region D) are distributed at the boundaries of the HEAMox (region A) matrix. The matrix powder is uniformly distributed in the composite, and they are connected to each other or bonded by the added phases. The interconnected HEAMox powder forms the matrix of the self-lubricating composite. The Ni coating effectively inhibits the reaction between MoS_2_ and HEAMox matrix powder while enhancing their bonding strength. Therefore, the composite may have excellent mechanical and tribological properties in a wide temperature range. As shown in the high-magnification micrographs of the matrix powder (a_1_, b_1_ and c_1_), the microstructure shows obvious composition segregation characteristics. With the increase in Mo element content, the volume fraction of white precipitated phase increases, and the volume fraction of black precipitated phase decreases. EDS analysis shows that the black precipitated phase has a high content of Cr element, the white precipitated phase has a high content of Mo element, and the light gray matrix has high contents of Fe, Co and Ni elements. Combined with X-ray diffraction analysis results, the white precipitated phase is σ phase, and the black precipitated phase is Cr-rich face-centered cubic phase. The lattice constant of the Cr-rich face-centered cubic phase is very close to that of the main face-centered cubic phase of CoCrFeNi, so it is difficult to detect the Cr-rich face-centered cubic phase with a conventional X-ray diffractometer [17].

### 3.2. Mechanical Properties

**Figure 3:**
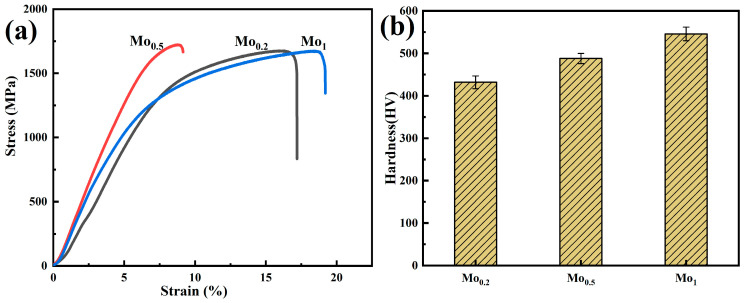
Mechanical properties of composites with different Mo contents: (**a**) Compressive stress–strain curves; (**b**) Vickers hardness.

Figure 3 and Table 1 show the compressive stress–strain curves and related mechanical properties of composites. The results show that the hardness, yield strength, and plastic strain of Mo0.2 are 432 HV, 445 MPa, and 15%, respectively. When the Mo content increases from 0.2 to 0.5, the hardness increases from 432 HV to 488 HV, the yield strength increases from 445 MPa to 502 MPa, while the plastic strain decreases from 15% to 10%. The reason for the increase in strength and hardness of Mo0.5 is that the Mo atoms are partially dissolved in the HEA matrix, which increases the lattice distortion of the matrix, and hinders the movement of dislocations through the dragging effect of solute atoms, resulting in a solid solution strengthening effect [20,21].

In addition, Mo and Cr elements form hard σ phase through in situ reaction, and the second-phase strengthening effect of hard σ phase helps to improve the yield strength and hardness of the alloy. Although the increase in Mo content leads to an increase in the content of hard σ phase, the FCC phase has good plasticity, and the hard σ phase has high hardness and poor plasticity. During deformation, it is difficult for the σ phase and FCC phase to deform simultaneously, which is the reason for the significant decrease in the plasticity of the HEA. The Mo1 composite has the highest hardness of 546 HV, which is 26% higher than that of Mo0.2, but its plastic strain is 17%, which is close to that of Mo0.2. The reason for the increase in hardness and plasticity is that the powder size of the Mo1 matrix alloy is 5–27 μm, which is much smaller than that of Mo0.2 and Mo0.5 matrix powders. Fine powder can improve the mechanical and tribological properties and structural uniformity of the material. According to the traditional sintering theory, compared with coarse particles, fine powder will generate greater sintering driving force, promote grain boundary diffusion, and the obtained composite has higher density and hardness.

### 3.3. Tribological Properties

**Figure 4:**
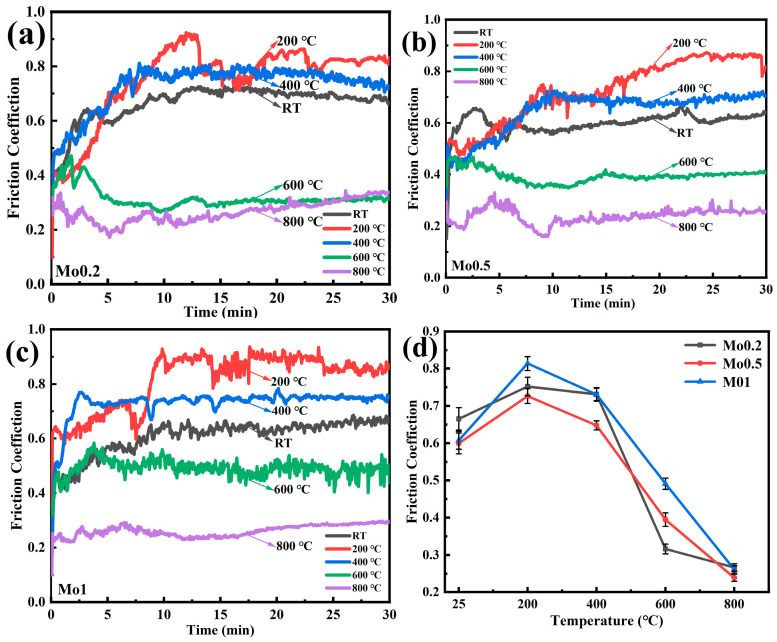
COF profiles of composites with varying Mo contents: (**a**) Mo0.2, (**b**) Mo0.5, (**c**) Mo1, and (**d**) temperature-dependent average COFs.

Figure 4a–c present the COF curves of Mo0.2, Mo0.5, and Mo1 at different test temperatures, respectively. At 200 °C, Mo0.2 exhibits the highest COF with the most pronounced fluctuations. Under steady-state wear conditions, the COF curves at 200 °C and 400 °C are higher than those at RT, whereas the COFs at 600 °C and 800 °C are comparable and lower than the room-temperature levels. For Mo0.5, COF evolution at room temperature, 200 °C, and 400 °C follows trends similar to those of Mo0.2. At 600 °C, the COF curve stabilizes with a significant reduction in magnitude. As the temperature further increases to 800 °C, the COF decreases further. Mo1 shows similar COF trends to Mo0.5 across all test temperatures, indicating consistent tribological behavior between these compositions.

Figure 4d displays the variations in the average coefficient of friction with temperature for composites with varying molybdenum concentrations. The analytical results show that Mo0.2 has a room temperature average COF of 0.66, and its maximum value of 0.75 occurs at 200 °C. As the temperature rises from 400 °C to 800 °C, the average COF gradually decreases from 0.73 to 0.21. Throughout the experimental temperature range, Mo0.5 exhibits COF evolution patterns consistent with those of Mo0.2. Although Mo0.5 has a higher average COF of 0.39 than Mo0.2 at 600 °C, it maintains lower values at all other test temperatures compared to Mo0.2.

Mo1 shows similar COF behavior to the aforementioned two composites. While the RT COF of Mo1 is approximately 0.60, similar to that of Mo0.5, its 800 °C value reaches 0.26, which is close to that of Mo0.2 and slightly higher than Mo0.5’s value of 0.23. All three composites reach peak COF values at 200 °C, after which the COF decreases systematically with increasing temperature until reaching minimum levels at 800 °C. This phenomenon may be attributed to the formation of different oxide species at different temperatures [6,22]. Except for the 600 °C data point, Mo0.5 consistently shows better friction reduction characteristics across the entire temperature range from ambient conditions to 800 °C, with its COF values lower than those of the other two composites.

**Figure 5:**
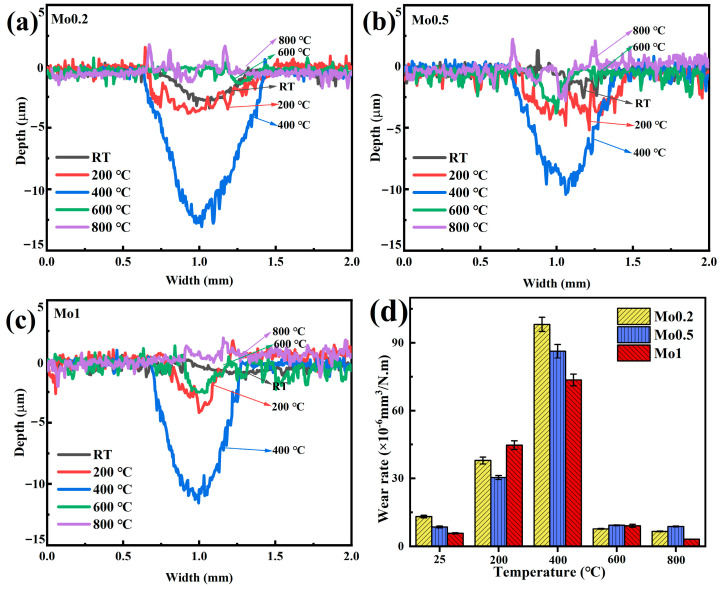
Two-dimensional surface profilometry of wear tracks in composites with varying Mo contents: (**a**) Mo0.2, (**b**) Mo0.5, (**c**) Mo1, and (**d**) temperature-dependent average WR.

Two-dimensional profiles of wear scars for Mo0.2, Mo0.5, and Mo1 composites across the tested temperature range are depicted in Figure 5a–c. Surface irregularities observed in all profiles originate from cyclic debris detachment and localized fracture. At 400 °C, where wear scars are most severe, Mo0.2 exhibits a depth of 13.3 μm and a width of 0.81 mm, Mo0.5 a depth of 9.8 μm and a width of 0.71 mm, and Mo1 a depth of 11.9 μm and a width of 0.63 mm. Both the depth and width of the wear scars decrease progressively with increasing temperature, reaching minima at 800 °C. The WR of the composites exhibit a positive correlation with the wear scar dimensions, as increased width and depth directly reflect enhanced material removal [3].

Figure 5d illustrates the evolution of wear rates for composites across different test temperatures. All three composites exhibit a substantial increase in WR from RT to 400 °C, reaching maxima at 400 °C, followed by a significant decrease at higher temperatures. For Mo0.2, the wear rate increases from 13.1 × 10^−6^ mm^3^/N·m at RT to 98.1 × 10^−6^ mm^3^/N·m at 400 °C, then decreases to 6.6 × 10^−6^ mm^3^/N·m at 800 °C. Similarly, Mo0.5 shows a progressive increase from 8.0 × 10^−6^ mm^3^/N·m at RT to 86.3 × 10^−6^ mm^3^/N·m at 400 °C, followed by a continuous decline to 8.75 × 10^−6^ mm^3^/N·m at 800 °C. Throughout the entire temperature range, Mo0.5 maintains lower WR compared to Mo0.2. For Mo1, the wear rate is 5.7 × 10^−6^ mm^3^/N·m at RT, reaches a peak of 73.5 × 10^−6^ mm^3^/N·m at 400 °C, and subsequently decreases to 3.15 × 10^−6^ mm^3^/N·m at 800 °C. Across the temperature range from RT to 800 °C, all composites exhibit WR below 1.0 × 10^−5^ mm^3^/N·m indicative of excellent wear resistance.

**Figure 6:**
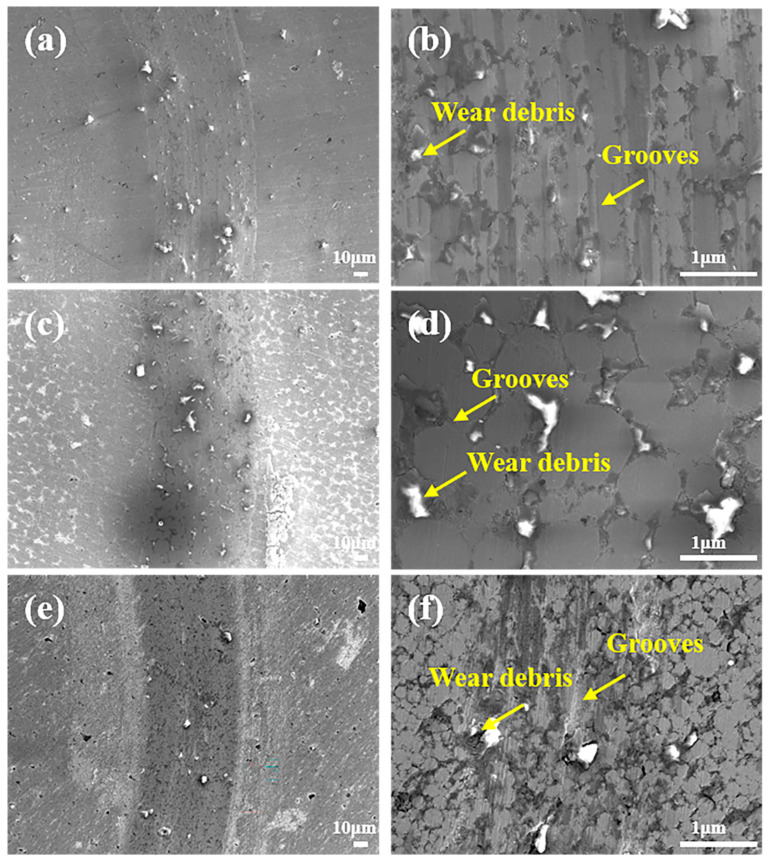
Wear scar morphology of composites with varying Mo contents after RT friction tests: (**a**,**b**) Mo0.2, (**c**,**d**) Mo0.5, and (**e**,**f**) Mo1.

As depicted in Figure 6a–f, the width of wear scars at RT gradually decreases with increasing Mo content, indicating that the formation of the hard σ-phase contributes to improved wear resistance, which is consistent with the wear rate trends shown in Figure 5d. Figure 6b reveals shallow grooves and minimal debris on the relatively smooth surface of Mo0.2, indicating that abrasive wear is the dominant mechanism. For Mo0.5 (Figure 6c,d), the reduced scar width is accompanied by shallower grooves and less debris accumulation compared to composites with lower Mo content, though abrasive wear remains the primary mechanism. As shown in Figure 6e,f, Mo1 exhibits the narrowest scars, flattest surfaces, and the least debris accumulation among all compositions, with abrasive wear still dominating. These observations collectively confirm that both solid solution strengthening and second-phase strengthening induced by the hard σ-phase significantly enhance the hardness and strength of the composites. At lower temperatures, abrasive wear predominantly governs the wear behavior, attributed to limited adhesive interactions between dissimilar counterface materials (e.g., ceramics and metals) [13,23].

**Figure 7:**
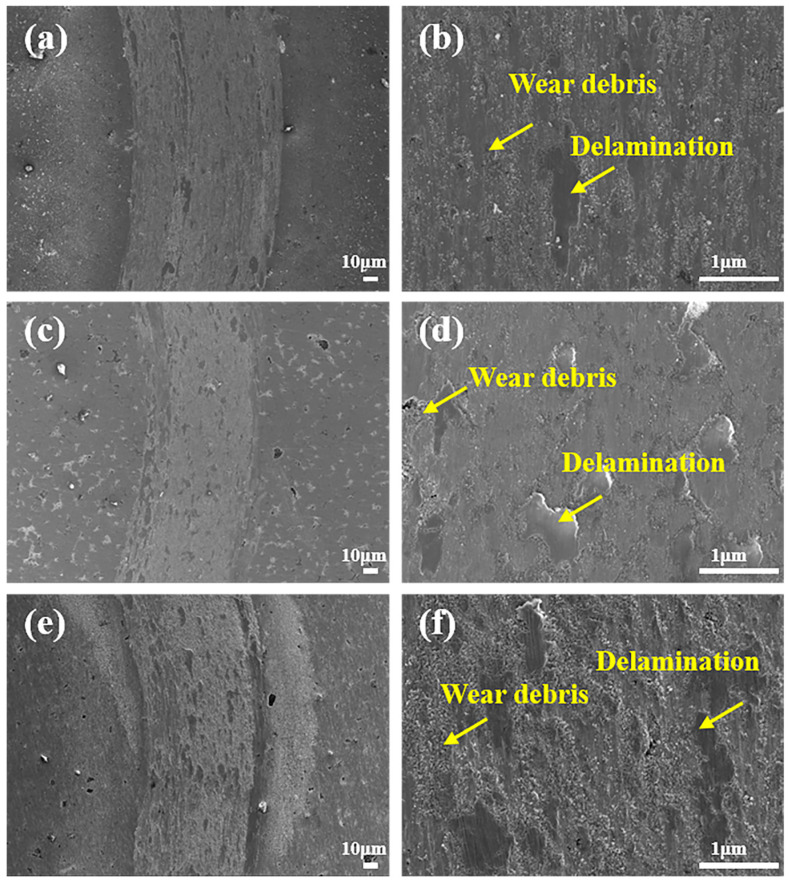
Wear scar morphology of composites with different Mo contents after friction tests at 200 °C: (**a**,**b**) Mo0.2, (**c**,**d**) Mo0.5, and (**e**,**f**) Mo1.

Figure 7 presents the wear scar morphologies of composites with varying Mo contents at 200 °C. The scar width first decreases then increases with increasing Mo content. Due to high-temperature softening effects, all composites exhibit significantly wider scars compared to those at RT, with distinct morphological differences. As shown in Figure 7b, Mo0.2 displays rough scar surfaces featuring grooves, wear debris, and minor spalling, indicating that both abrasive and adhesive wear are the dominant mechanisms. Figure 7d reveals that Mo0.5 exhibits smoother surfaces with shallower grooves but increased spalling compared to Mo0.2, indicating a more significant contribution from adhesive wear. Figure 7e,f show that Mo1 exhibits the widest scars with substantial debris accumulation along edges, rough surfaces with abundant debris and spalling, where both abrasive and adhesive wear dominate. Surface grooves arise from micro-cutting action by the Si_3_N_4_ counterface ball, while wear debris consists of fragmented particles generated during micro-cutting. Surface spalling results from material transfer phenomena. Elevated temperatures reduce the hardness of the materials, making the composites more susceptible to cutting by the harder Si_3_N_4_ counterface. This increased surface roughness inhibits effective spreading of solid lubricants to form continuous lubricating films, thereby increasing the COF and WR. These observations are consistent with the results presented in Figure 4d and Figure 5d [12].

**Figure 8:**
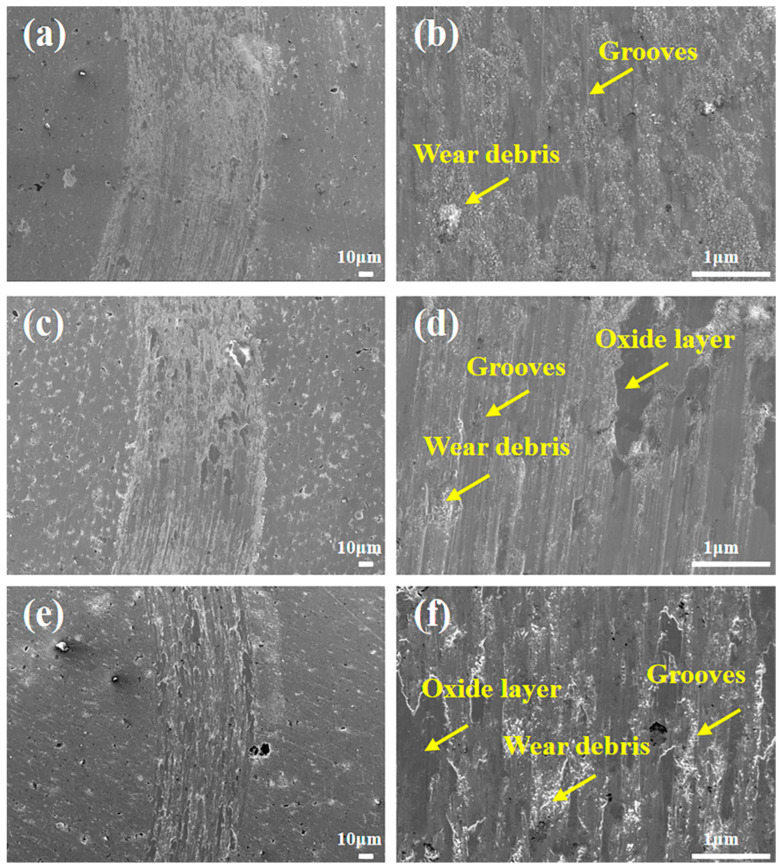
Wear scar morphology of composites with varying Mo contents after friction tests at 400 °C: (**a**,**b**) Mo0.2, (**c**,**d**) Mo0.5, and (**e**,**f**) Mo1.

Figure 8 illustrates the wear scar morphologies of composites tested at 400 °C. All composites exhibit markedly broader wear scars compared to those at 200 °C, with rough surfaces featuring grooves, wear debris, and discontinuous dark oxide layers, consistent with dominant abrasive and oxidative wear mechanisms. Increasing Mo content enhances hardness, leading to gradual smoothing of scar surfaces and reduced scar width. Notably, the peak WR observed at 400 °C for all compositions arises from thermally induced softening, which increases susceptibility to cutting by the counterface ball [24]. The friction-reducing mechanism involves the extrusion of soft Ag under the combined action of frictional stress and frictional heat; the extruded Ag migrates to the worn surfaces and cooperates with layered MoS_2_ to provide lubrication. However, surface roughness hinders the formation of continuous lubricating films. Consequently, although the friction coefficients at 400 °C are lower than those at 200 °C, the friction-reducing effect remains constrained [9].

**Figure 9:**
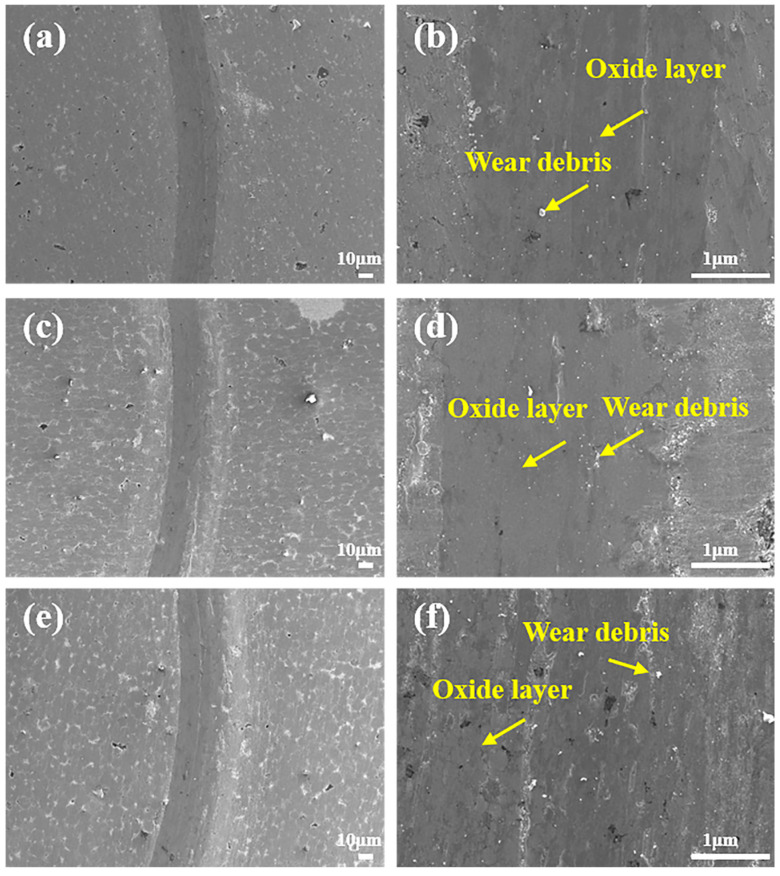
Wear scar morphology of composites with varying Mo contents after friction tests at 600 °C: (**a**,**b**) Mo0.2, (**c**,**d**) Mo0.5, and (**e**,**f**) Mo1.

Figure 9 presents the wear scar morphologies of composites with different Mo contents at 600 °C. It can be observed that compared with the wear scar width at 400 °C, the wear scar widths of the three composites are basically consistent and all significantly narrowed, with the WR reduced by an order of magnitude compared to that at 400 °C, which is consistent with Figure 5d. Different from the rough wear morphology at 400 °C, as the temperature increases, the higher test temperature and sliding wear process jointly intensify the oxidation behavior of the wear surface, leading to the formation of various metal oxides on the wear surface [6]; however, the oxides have insufficient compactness, resulting in fine and loose wear debris distributed on the wear scars, and the wear mechanism is dominated by oxidative wear at this time. At this temperature, both Ag and Mo elements are oxidized, and their oxides further generate silver molybdate through tribochemical reactions; the silver molybdate with a layered structure can form a lubricating film on the friction surface at high temperature, which significantly reduces the friction coefficient and wear rate [7,25]. Thus, the good tribological properties of composites with different Mo contents at 600 °C may be attributed to the initial formation of the silver molybdate phase, while the relatively rough wear surface prevents the formation of a continuous and compact oxide layer, resulting in lower COF and WR at 600 °C than those at 800 °C, which is consistent with the descriptions in Figure 4 and Figure 5.

**Figure 10:**
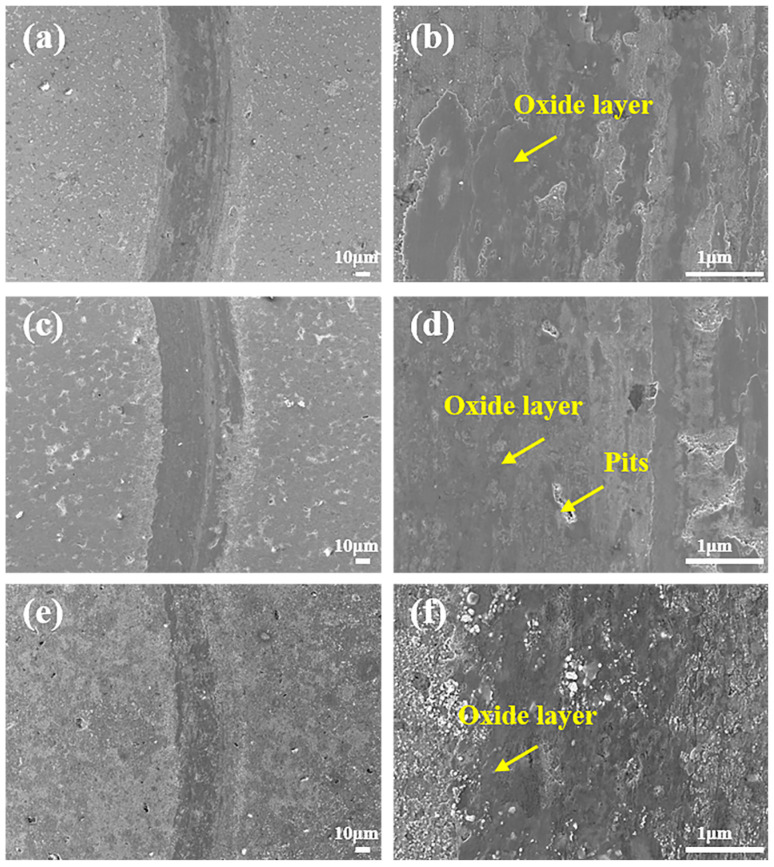
Wear scar morphology of composites with varying Mo contents after friction tests at 800 °C: (**a**,**b**) Mo0.2, (**c**,**d**) Mo0.5, and (**e**,**f**) Mo1.

Figure 10 shows the wear surface morphologies of the composites at 800 °C. At such elevated temperatures, oxidation processes dominate tribological behavior, with oxide layers formed on all worn surfaces [12]. Scar width first increases and then decreases with increasing Mo content. For Mo0.2 (Figure 10b), an incoherent glaze layer forms, offering limited improvement in wear resistance, with oxidative wear as the dominant mechanism. Mo0.5 (Figure 10d) exhibits expanded glaze coverage but contains pits arising from adhesive interactions between the composite and the Si_3_N_4_ ball, inducing material detachment and tear pit formation; this composition undergoes combined oxidative and adhesive wear. Notably, Mo1 (Figure 10f) achieves the narrowest scar width, with abundant loose white oxide particles distributed outside the scars. Within the scars, the synergistic effect of frictional heat and normal pressure compacts most oxide particles into a continuous, dense glaze layer that fully covers the surface. This integrated glaze layer inhibits direct contact between the Si_3_N_4_ ball and the composite substrate, significantly reducing interfacial shear. Consequently, the COF and WR reach optimal values of 0.26 and 3.15 × 10^−6^ mm^3^/N·m, respectively, demonstrating exceptional friction reduction and wear resistance via oxidative wear mechanisms [2,12].

To further verify the excellent mechanical properties of the Mo1 composite, Table 2 compares the performance data of several composite materials. The results show that the Mo1 composite has the highest hardness, reaching 546 HV, and compared with the CoCrFeNi-Ni/MoS_2_-Ag-Cr_2_O_3_ composite with the same additive components, it maintains a similar level of yield strength and plastic strain. According to the basic laws of material tribology, generally, the higher the material hardness, the stronger its ability to resist surface plastic deformation, scratching and adhesive wear, and the lower the WR [5]. The wear rate of the Mo1 composite in the temperature range from RT to 800 °C is one order of magnitude lower than that of the CoCrFeNi-Ag-Mo composite. Among them, at 800 °C, the COF is as low as 0.26 and the WR is only 3.15 × 10^−6^ mm^3^/N·m. In conclusion, the Mo1 composite studied in this paper possesses both excellent mechanical properties and tribological properties.

**Figure 11:**
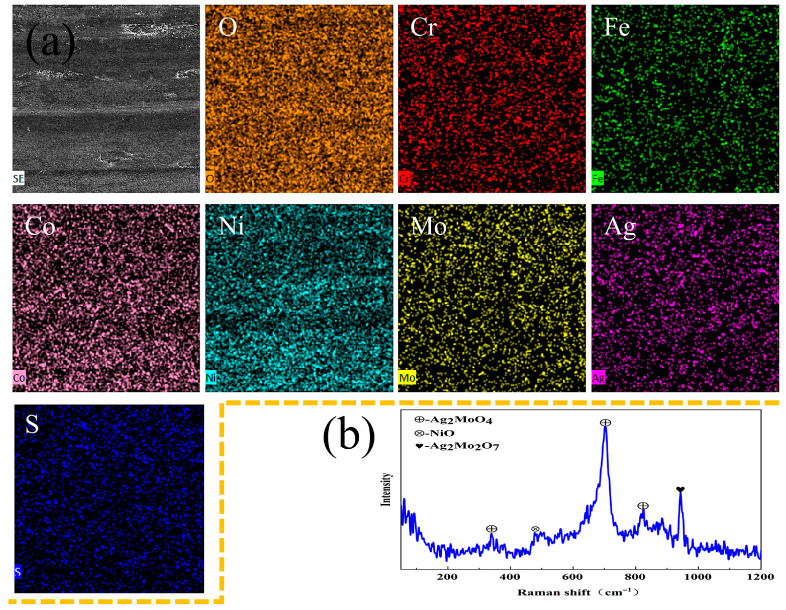
SEM image and element mapping (**a**) as well as Raman spectrum (**b**) of the wear scar on Mo1 composite after friction testing at 800 °C.

As indicated by the previous friction and wear data, only the Mo1 composite exhibits both excellent friction-reducing and wear-resistant tribological properties at 800 °C, which is obviously closely related to the continuous, dense glaze layer that forms on the worn surface and fully covers it (Figure 10e,f). To further clarify the specific formation mechanism of the glaze layer, element mapping analysis was performed on the wear scar surface (Figure 11a). The results clearly show that Ag, Mo, and O three elements are uniformly distributed on the worn surface. This directly confirms that oxidative wear dominates the wear process of the composite at 800 °C, and the lubricating film formed by such oxidation products is the core guarantee for its tribological performance.

To further identify the composition of the glaze layer, Raman spectroscopy was used for accurate characterization of the worn surface composition (Figure 11b). The analysis results show that the oxide glaze layer formed on the worn surface is mainly composed of nickel oxide (NiO) and multiple silver molybdate phases (Ag_2_MoO_4_, Ag_2_Mo_4_O_13_, and Ag_2_Mo_2_O_7_). These silver molybdate phases exert a synergistic lubricating effect at high temperatures, significantly improving the tribological performance of the composite. Furthermore, the continuous and dense oxide glaze layer (lubricating film) can effectively inhibit direct contact between the composite matrix and the Si_3_N_4_ counterface ball [7]. This protective effect further enhances the high-temperature tribological stability of the material, and ultimately directly accounts for the excellent tribological performance of the Mo1 composite at 800 °C [25,26].

## 4. Conclusions

Self-lubricating HEAMo_x_-Ni/MoS_2_-Ag-Cr_2_O_3_ composites were fabricated by SPS. The influence of Mo content on the microstructure, mechanical properties, and tribological performance of the composites were investigated, and the friction and wear mechanisms over a wide temperature range were analyzed. The main conclusions are as follows:

1. The composite Mo0.2 has an FCC lattice and consists of Ag, Ni, MoS_2_ and Cr_2_O_3_. Increasing the Mo content promotes the formation of the hard σ phase and induces significant lattice distortion due to Mo solid solution.
2. Solid solution strengthening by Mo atoms and the formation of the hard σ phase significantly enhance the hardness of the composites. As the Mo content increases from 0.2 to 0.5, the hardness rises from 432 HV to 546 HV.
3. The Mo0.2 composite exhibits favorable friction-reducing properties over a wide temperature range, particularly achieving a low COF of 0.23 at 800 °C. All composites with varying Mo contents exhibit wear rates lower than 10^−5^ mm^3^/N·m from RT to 800 °C. Notably, the Mo1 composite demonstrates an exceptionally low wear rates of 3.15 × 10^−6^ mm^3^/N·m at 800 °C.

## Figures and Tables

**Table 1:** Mechanical properties of composites with varying Mo contents.

Composites	Hardness (HV)	Yield Strength (MPa)	Plasticity Strain (%)
Mo0.2	432	445	15
Mo0.5	488	502	10
Mo1	546	443	17

**Table 2:** Detailed comparison of mechanical properties and tribological parameters for composites.

Composites	Mechanical Properties			COF			WR (×10^−6^ mm^3^/N·m)		
	Hardness (HV)	Yield Strength (MPa)	Plastic Strain (%)	RT	400 °C	800 °C	RT	400 °C	800 °C
CoCrFeNi-Ag-Mo [6]	238	435	>45	0.49	0.35	0.32	170	220	32
CoCrFeNi-Ni/MoS_2_-Ag-Cr_2_O_3_ [26]	382	430	20	0.69	0.68	0.45	9.9	47.8	3.3
CoCrFeNi-Ag-CaF_2_/BaF_2_ [16]	151	468	15.8	0.24	0.26	0.20	42	88	59
CoCrFeNi-Ni/MoS_2_-Ag [26]	342	371	25	0.55	0.51	0.44	20.6	83.3	9.8
CoCrFeNiMo_1_-Ni/MoS_2_-Ag-Cr_2_O_3_	546	443	17	0.61	0.73	0.26	5.7	73.5	3.1

## Data Availability

The original contributions presented in this study are included in the article. Further inquiries can be directed to the corresponding author.
